# Provider adherence to clinical care recommendations for infants and children who died in seven low- and middle-income countries in the Child Health and Mortality Prevention Surveillance (CHAMPS) network

**DOI:** 10.1016/j.eclinm.2023.102198

**Published:** 2023-08-31

**Authors:** Chris A. Rees, Kitiezo Aggrey Igunza, Zachary J. Madewell, Victor Akelo, Dickens Onyango, Shams El Arifeen, Emily S. Gurley, Mohammad Zahid Hossain, Afruna Rahman, Muntasir Alam, J. Anthony G. Scott, Nega Assefa, Lola Madrid, Anteneh Belachew, Haleluya Leulseged, Karen L. Kotloff, Samba O. Sow, Milagritos D. Tapia, Adama Mamby Keita, Diakaridia Sidibe, Antonio Sitoe, Rosauro Varo, Sara Ajanovic, Quique Bassat, Inácio Mandomando, Beth A. Tippett Barr, Ikechukwu Ogbuanu, Carrie Jo Cain, Ima-Abasi Bassey, Ronita Luke, Khadija Gassama, Shabir Madhi, Ziyaad Dangor, Sana Mahtab, Sithembiso Velaphi, Jeanie du Toit, Portia C. Mutevedzi, Dianna M. Blau, Robert F. Breiman, Cynthia G. Whitney, Child Health and Mortality Prevention Surveillance Network, Fatima Solomon, Gillian Sorour, Hennie Lombaard, Jeannette Wadula, Karen Petersen, Martin Hale, Nelesh P. Govender, Peter J. Swart, Sanjay G. Lala, Sithembiso Velaphi, Richard Chawana, Yasmin Adam, Amy Wise, Ashleigh Fritz, Nellie Myburgh, Pedzisai Ndagurwa, Cleopas Hwinya, Sanwarul Bari, Shahana Parveen, Mohammed Kamal, A.S.M. Nawshad Uddin Ahmed, Mahbubul Hoque, Saria Tasnim, Ferdousi Islam, Farida Ariuman, Mohammad Mosiur Rahman, Ferdousi Begum, K. Zaman, Mustafizur Rahman, Dilruba Ahmed, Meerjady Sabrina Flora, Tahmina Shirin, Mahbubur Rahman, Joseph Oundo, Alexander M. Ibrahim, Fikremelekot Temesgen, Tadesse Gure, Addisu Alemu, Melisachew Mulatu Yeshi, Mahlet Abayneh Gizaw, Stian Orlien, Solomon Ali, Peter Otieno, Peter Nyamthimba Onyango, Janet Agaya, Richard Oliech, Joyce Akinyi Were, Dickson Gethi, Sammy Khagayi, George Aol, Thomas Misore, Harun Owuor, Christopher Mugah, Bernard Oluoch, Christine Ochola, Sharon M. Tennant, Carol L. Greene, Ashka Mehta, J. Kristie Johnson, Brigitte Gaume, Rima Koka, Karen D. Fairchild, Diakaridia Kone, Sharon M. Tennant, Ashka Mehta, Doh Sanogo, Uma U. Onwuchekwa, Nana Kourouma, Seydou Sissoko, Cheick Bougadari Traore, Jane Juma, Kounandji Diarra, Awa Traore, Tiéman Diarra, Kiranpreet Chawla, Tacilta Nhampossa, Zara Manhique, Sibone Mocumbi, Clara Menéndez, Khátia Munguambe, Ariel Nhacolo, Maria Maixenchs, Andrew Moseray, Fatmata Bintu Tarawally, Martin Seppeh, Ronald Mash, Julius Ojulong, Babatunde Duduyemi, James Bunn, Alim Swaray-Deen, Joseph Bangura, Amara Jambai, Margaret Mannah, Okokon Ita, Cornell Chukwuegbo, Sulaiman Sannoh, Princewill Nwajiobi, Dickens Kowuor, Erick Kaluma, Oluseyi Balogun, Solomon Samura, Samuel Pratt, Francis Moses, Tom Sesay, James Squire, Joseph Kamanda Sesay, Osman Kaykay, Binyam Halu, Hailemariam Legesse, Francis Smart, Sartie Kenneh, Soter Ameh, Sartie Kenneh, Jana Ritter, Tais Wilson, Jonas Winchell, Jakob Witherbee, Navit T. Salzberg, Jeffrey P. Koplan, Margaret Basket, Ashutosh Wadhwa, Kyu Han Lee, Valentine Wanga, Roosecelis Martines, Shamta Warang, Maureen Diaz, Jessica Waller, Shailesh Nair, Lucy Liu, Courtney Bursuc, Kristin LaHatte, Sarah Raymer, John Blevins, Solveig Argeseanu, Kurt Vyas, Manu Bhandari

**Affiliations:** a Department of Pediatrics, Emory University School of Medicine, Atlanta, GA, United States of America; b Children's Healthcare of Atlanta, Atlanta, GA, United States of America; c Kenya Medical Research Institute-Center for Global Health Research, Kisumu, Kenya; d Center for Global Health, Centers for Disease Control and Prevention, Atlanta, GA, United States of America; e Centers for Disease Control and Prevention-Kenya, Kisumu, Kenya; f Kisumu County Department of Health, Kisumu, Kenya; g International Centre for Diarrhoeal Disease Research Bangladesh (icddr,b), Dhaka, Bangladesh; h Department of Epidemiology, Johns Hopkins Bloomberg School of Public Health, Baltimore, MD, United States of America; i London School of Hygiene and Tropical Medicine, Keppel Street, London, UK; j College of Health and Medical Sciences, Haramaya University, Harar, Ethiopia; k Hararghe Health Research, Haramaya University, Ethiopia; l Department of Pediatrics, Center for Vaccine Development and Global Health, University of Maryland School of Medicine, Baltimore, MD, United States of America; m Centre pour le Développement des Vaccins-Mali, Bamako, Mali; n Centro de Investigação em Saúde de Manhiça [CISM], Maputo, Mozambique; o ISGlobal - Hospital Clínic, Universitat de Barcelona, Barcelona, Spain; p ICREA, Pg. Lluís Companys 23, 08010, Barcelona, Spain; q Pediatrics Department, Hospital Sant Joan de Déu, Universitat de Barcelona, Esplugues, Barcelona, Spain; r Consorcio de Investigación Biomédica en Red de Epidemiología y Salud Pública (CIBERESP), Madrid, Spain; s Instituto Nacional de Saúde, Ministério de Saúde, Maputo, Moçambique; t Nyanja Health, Salima, Malawi; u Crown Agents, Freetown, Sierra Leone; v World Hope International, Freetown, Sierra Leone; w Ministry of Health and Sanitation, Freetown, Sierra Leone; x South African Medical Research Council Vaccines and Infectious Diseases Analytics Research Unit, University of the Witwatersrand, Johannesburg, South Africa; y Department of Paediatrics and Child Health, Chris Hani Baragwanath Academic Hospital, University of the Witwatersrand, Johannesburg, South Africa; z Global Health Institute, Emory University, Atlanta, GA, United States of America; aa Hubert Department of Global Health, Rollins School of Public Health, Emory University, Atlanta, GA, United States of America

**Keywords:** Childhood, Mortality, Clinical care, Guideline adherence

## Abstract

**Background:**

Most childhood deaths globally are considered preventable through high-quality clinical care, which includes adherence to clinical care recommendations. Our objective was to describe adherence to World Health Organization recommendations for the management of leading causes of death among children.

**Methods:**

We conducted a retrospective, descriptive study examining clinical data for children aged 1–59 months who were hospitalized and died in a Child Health and Mortality Prevention Surveillance (CHAMPS) catchment, December 2016–June 2021. Catchment areas included: Baliakandi and Faridpur, Bangladesh; Kersa, Haramaya, and Harar, Ethiopia; Kisumu and Siaya, Kenya; Bamako, Mali; Manhiça and Quelimane, Mozambique; Makeni, Sierra Leone; Soweto, South Africa. We reviewed medical records of those who died from lower respiratory tract infections, sepsis, malnutrition, malaria, and diarrheal diseases to determine the proportion who received recommended treatments and compared adherence by hospitalization duration.

**Findings:**

CHAMPS enrolled 460 hospitalized children who died from the leading causes (median age 12 months, 53.0% male). Median hospital admission was 31 h. There were 51.0% (n = 127/249) of children who died from lower respiratory tract infections received supplemental oxygen. Administration of intravenous fluids for sepsis (15.9%, n = 36/226) and supplemental feeds for malnutrition (14.0%, n = 18/129) were uncommon. There were 51.4% (n = 55/107) of those who died from malaria received antimalarials. Of the 80 children who died from diarrheal diseases, 76.2% received intravenous fluids. Those admitted for ≥24 h more commonly received antibiotics for lower respiratory tract infections and sepsis, supplemental feeds for malnutrition, and intravenous fluids for sepsis than those admitted <24 h.

**Interpretation:**

Provision of recommended clinical care for leading causes of death among young children was suboptimal. Further studies are needed to understand the reasons for deficits in clinical care recommendation adherence.

**Funding:**

10.13039/100000865Bill & Melinda Gates Foundation.

**Research in context**

**Evidence before this study**

More than 80% of the 5.2 million annual deaths among children aged <5 years occur in sub-Saharan Africa and South Asia. Most of these deaths are considered to be preventable through timely access to, and the provision of, high quality clinical care, which includes adherence to clinical care guideline recommendations. Prior studies suggest that adherence to clinical care recommendations in several sites in sub-Saharan Africa and Southeast Asia for children who survived common illnesses requiring hospitalization has varied; however, an understanding of gaps in clinical care guideline recommendation adherence among infants and children who died is lacking.

**Added value of this study**

Adherence to clinical care recommendations including supplemental oxygen for children with lower respiratory tract infections, administration of intravenous fluids for sepsis, supplemental feeds for severe acute malnutrition, antimalarial medications for malaria, and intravenous fluids for diarrheal diseases was generally suboptimal among 460 hospitalized children aged 1–59 months who died while hospitalized in six sites in sub-Saharan Africa and Bangladesh. However, adherence to key recommendations improved in cases in which the hospitalization lasted ≥24 h and in cases in which clinicians made a diagnosis antemortem that was concordant with postmortem diagnoses. These findings can be used to inform health systems changes to improve the availability of critical diagnostics and therapeutics.

**Implications of all the available evidence**

Adherence to clinical care recommendations for leading causes of death among young children was suboptimal in seven sites with high childhood mortality rates. Further studies are needed to understand the reasons for deficits in clinical care recommendation adherence. Additionally, efforts are urgently needed to promote adherence to clinical care guideline recommendations in order to reduce childhood mortality in sub-Saharan Africa and Bangladesh.

## Introduction

Expanded treatment availability, improvements in country-wide and individual socioeconomic status, and widespread vaccination programs have contributed to dramatic reductions globally in mortality among children aged <5 years.^1^^,^^2^ However, progress in reducing mortality among children aged <5 years has been geographically unequal. More than >80% of deaths among children aged <5 years occur in sub-Saharan Africa and South Asia and rates of mortality among children aged <5 years remain as high as 74 per 1,000 live births in sub-Saharan Africa and as high as 37 per 1,000 live births in South Asia.^3^, ^4^, ^5^ Most of these deaths are considered to be preventable through access to, and the provision of, high quality clinical care.^6^

Prior studies addressing mortality among children aged <5 years have focused on patient-level factors and have called for increasing access to healthcare facilities to reduce mortality in this age group.^7^, ^8^, ^9^, ^10^, ^11^ The actual quality of clinical care children receive once treated in healthcare facilities in sub-Saharan Africa and South Asia has received much less attention.^12^ However, understanding specific gaps in clinical care may allow for the development of targeted clinical interventions to reduce mortality in this age group in resource-limited settings.

The World Health Organization (WHO) and Ministries of Health in many countries have developed clinical care recommendations for common conditions among children to promote the provision of standardized, quality clinical care.^13^ Prior studies suggest that implementing standardized, evidence-based recommendations in hospital settings may reduce childhood mortality in resource-limited settings.^14^, ^15^, ^16^ However, adherence to clinical care recommendations in several sites in sub-Saharan Africa, Bangladesh, and Pakistan has varied,^17^ and an understanding of potential gaps in recommendation adherence among infants and children who died is lacking.

Our objective was to describe provider adherence to WHO clinical care recommendations for the five leading causes of death for infants and children who died in hospitals within high-mortality areas in seven countries.

## Methods

### Study design

We conducted a retrospective, descriptive study that examined clinical data for infants and children who died at seven sites in the Child Health and Mortality Prevention Surveillance (CHAMPS) network.

### Ethics

Ethical clearance was granted by each site's respective ethical review board as well as the institutional review board at Emory University Rollins School of Public Health.

### Study setting

The CHAMPS network was established in 2015 and generates detailed and longitudinal information on causes of death among stillbirths, neonates, infants, and children aged <5 years in regions with high childhood mortality rates. Such detailed and reliable data on specific causes of death developed by CHAMPS are crucial to develop targeted interventions more effectively to reduce mortality among children aged <5 years.^18^ CHAMPS sites were selected because they were regions with <5 mortality rates of >50 per 1,000 live births, had established surveillance track records, and agreed to a common, multisite protocol and to share data globally in real time.^19^^,^^20^ Catchment areas include: Baliakandi and Faridpur, Bangladesh; Kersa, Haramaya, and Harar, Ethiopia; Kisumu and Siaya, Kenya; Bamako, Mali; Manhiça and Quelimane, Mozambique; Makeni, Sierra Leone; Soweto, South Africa. CHAMPS staff in each of these areas conduct surveillance in both healthcare facilities, including national referral hospitals and district hospitals, and the community for stillbirths and child deaths. Stillbirths and neonates aged 0–28 days were not included in this study because clinical care recommendations for this age group differ from those among infants and children.

Once identified, caregivers of deceased children are approached by CHAMPS staff to assess for willingness to consent. Deaths identified within 24 h (up to 72 h if refrigerated) are eligible for minimally invasive tissue sampling (MITS), clinical record review, and verbal autopsy.^20^^,^^21^ MITS is an alternative to complete autopsy that has demonstrated acceptability in resource-limited settings as well as high concordance with complete diagnostic autopsy, which is the reference standard of determining postmortem causes of death.^19^^,^^22^, ^23^, ^24^, ^25^, ^26^ Deaths identified 24 h or later are eligible for verbal autopsy and clinical record review only. After caregivers provide informed written consent, CHAMPS staff perform MITS and collect demographic, verbal autopsy, microbiological, histopathological, and clinical information for each death. Body fluid and tissues from organs collected through MITS undergo a range of microbiology and histopathology tests.^19^ Verbal autopsies are conducted by CHAMPS staff, who are trained to conduct verbal autopsies using the standardized 2016 WHO Verbal Autopsy form.^27^ A panel of local experts (i.e., the Determination of Cause of Death [DeCoDe] panel) reviews all data and determines the causes of death by incorporating clinical, verbal autopsy, microbiological, and pathological data.^28^

### Data sources

Trained clinical staff employed by CHAMPS reviewed all available medical records (i.e., admission records, daily progress notes, laboratory and radiology results, and hospital registries) to extract detailed clinical data using standardized abstraction forms that include discrete variables for laboratory, radiologic, and therapeutic data. Additionally, the clinical abstraction form includes narrative fields in which CHAMPS team members at each site provided clinical summaries of all clinical care provided to an infant or child prior to death in both inpatient and outpatient settings as documented in the clinical records. We used both discrete variables and narrative fields to extract specific elements of clinical care based on lists of major diagnostics and primary therapeutics for the five leading causes of death that are recommended in the 2013 version of the WHO Pocket Book of Hospital Care for Children (2nd edition).^13^

The WHO Pocket Book of Hospital Care for Children recommendations have been adopted widely throughout sub-Saharan Africa and South Asia. Because CHAMPS includes seven sites in different countries and country-specific national recommendations may differ, we reviewed national guidelines for the included conditions.^29^, ^30^, ^31^, ^32^, ^33^, ^34^ However, because the major diagnostics and primary therapeutics did not differ for the included conditions, we restricted our analysis to a comparison of care received and that recommended by the WHO Pocket Book of Hospital Care for Children.

### Inclusion and exclusion criteria

We included all infants and children aged 1–59 months who enrolled in CHAMPS, were hospitalized, and were determined to have died from one or more of the five most common causes of death (i.e., lower respiratory infections, sepsis, severe acute malnutrition, malaria, and diarrheal diseases) by the DeCoDe panels according to the WHO International Statistical Classification of Diseases and Related Health Problems, Tenth Revision (ICD-10) and the WHO application of ICD-10 deaths during the perinatal period (ICD-PM) to assign causes of death.^35^^,^^36^ As multiple causes of death are often implicated in the causal chain of childhood mortality,^37^ we included infants and children who had any of these conditions in any position in the causal chain of death (i.e., the immediate cause of death, the underlying cause of death, or antecedent causes of death). We included deaths among infants and children that occurred from the inception of the CHAMPS network from December 2016 through June 2021. Deaths that occurred in the community were included if there was a hospital admission within seven days of the infant's or child's death. We excluded cases that had no hospital admission, those that were dead prior to or upon arrival at a healthcare facility, and those that did not have any clinical data available as it was not possible to determine the clinical care provided to these infants and children without existing records.

### Statistics

We calculated descriptive statistics for the demographics of the included deceased infants and children. We determined the proportion of deceased infants and children who received care that adhered to major diagnostic and therapeutic recommendations according to the WHO Pocket Book of Hospital Care for Children. In cases with >1 cause of death, the clinical care provided was compared to recommendations for each cause of death. As discrepancies between antemortem and postmortem diagnoses are common in resource-limited settings^38^ and because diagnostic clarity often comes with time, we conducted subanalyses to explore differences in administered diagnostics and therapeutics according to the WHO recommendations by the concordance of antemortem diagnoses (as documented in clinical records) with postmortem diagnoses and for the duration of hospital admission (i.e., <24 h vs. ≥24 h). We used mixed-effect univariable and multivariable logistic regression to identify factors associated with the administration of major therapeutics for lower respiratory tract infections, adjusting for age group (i.e., 1–11 months vs. 12–59 months), sex, time from admission to death, and concordant vs. discordant antemortem diagnosis compared to postmortem diagnosis, with site included as a random effect. In our multivariable models, we included variables that met our predetermined threshold of *P* ≤ 0.2 in the univariable comparisons. Variance inflation factors were used to assess collinearity among independent variables. Given that the administration of therapeutics may vary by both duration of hospital admission and concordance of antemortem diagnoses, effect modification was evaluated using an interaction term. We did not have adequate power to conduct similar multivariable models for sepsis, severe acute malnutrition, malaria, or diarrheal diseases. Proportions were compared using the Chi-square test. All analyses were conducted using the statistical software package R, version 4.2.3 (R Foundation for Statistical Computing, Vienna, Austria).

### Role of funding source

The funder had no role in the study design, data collection, analysis, results interpretation, writing of the report, or the decision to submit for publication.

## Results

During the study period, CHAMPS enrolled 651 infants and children who died from lower respiratory tract infections, sepsis, severe acute malnutrition, malaria, or diarrheal diseases; 501 (77.0%) were admitted to a hospital, 41 (6.3%) were dead prior to or upon arrival or had missing clinical records, and 460 (70.7%) had clinical records and causes of death determined by DeCoDe and, thus, were included in our analyses. Cases that were included had similar age and sex to those who were excluded and there were some differences in causes of death between included and excluded cases (Supplemental Table S1). The median age at the time of death was 12 months (interquartile range [IQR] 4, 23), and 53.0% (n = 244) were male (Table 1). The median duration of hospital admission was 31 h (IQR 6, 143) and 35.7% (n = 164) died <24 h after admission. Over half of the included infants and children were enrolled from two sites (South Africa 29.8% [n = 137], and Kenya 26.3% [n = 121]).

**Table 1:** Demographics of infants and children who died from the leading causes of death in the CHAMPS network (n = 460).

Characteristics	n (%)
**Patient Age, month (median, [IQR])**	12 (4, 23)
**Age at death**	
28 days old to less than 12 months old	231 (50.2)
12 months old to less than 60 months old	229 (49.8)
**Sex**	
Male	244 (53.0)
Female	216 (47.0)
**Site of death**	
Facility	424 (92.2)
Community	36 (7.8)
**Time of hospital death**	
Died in a facility <24 h	164 (35.7)
Died in a facility between 24 and 48 h	52 (11.3)
Died in a facility between 49 and 72 h	28 (6.1)
Died in a facility >72 h	175 (38.0)
Missing	41 (8.9)
**Country site**	
Bangladesh	3 (0.7)
Ethiopia	9 (2.0)
Kenya	121 (26.3)
Mali	33 (7.2)
Mozambique	74 (16.1)
Sierra Leone	83 (18.0)
South Africa	137 (29.8)
**Lower respiratory tract infections**	
Diagnosed by minimally invasive tissue sampling (MITS)	203 (50.6)
Concordant antemortem and postmortem diagnosis	80 (17.4)
**Sepsis**	
Diagnosed by MITS	193 (48.1)
Concordant antemortem and postmortem diagnosis	61 (13.3)
**Severe acute malnutrition**	
Diagnosed by MITS	127 (31.7)
Concordant antemortem and postmortem diagnosis	55 (12.0)
**Malaria**	
Diagnosed by MITS	107 (26.7)
Concordant antemortem and postmortem diagnosis	77 (16.7)
**Diarrheal diseases**	
Diagnosed by MITS	76 (19.0)
Concordant antemortem and postmortem diagnosis	57 (12.4)

### Lower respiratory tract infections

**Fig. 1:**
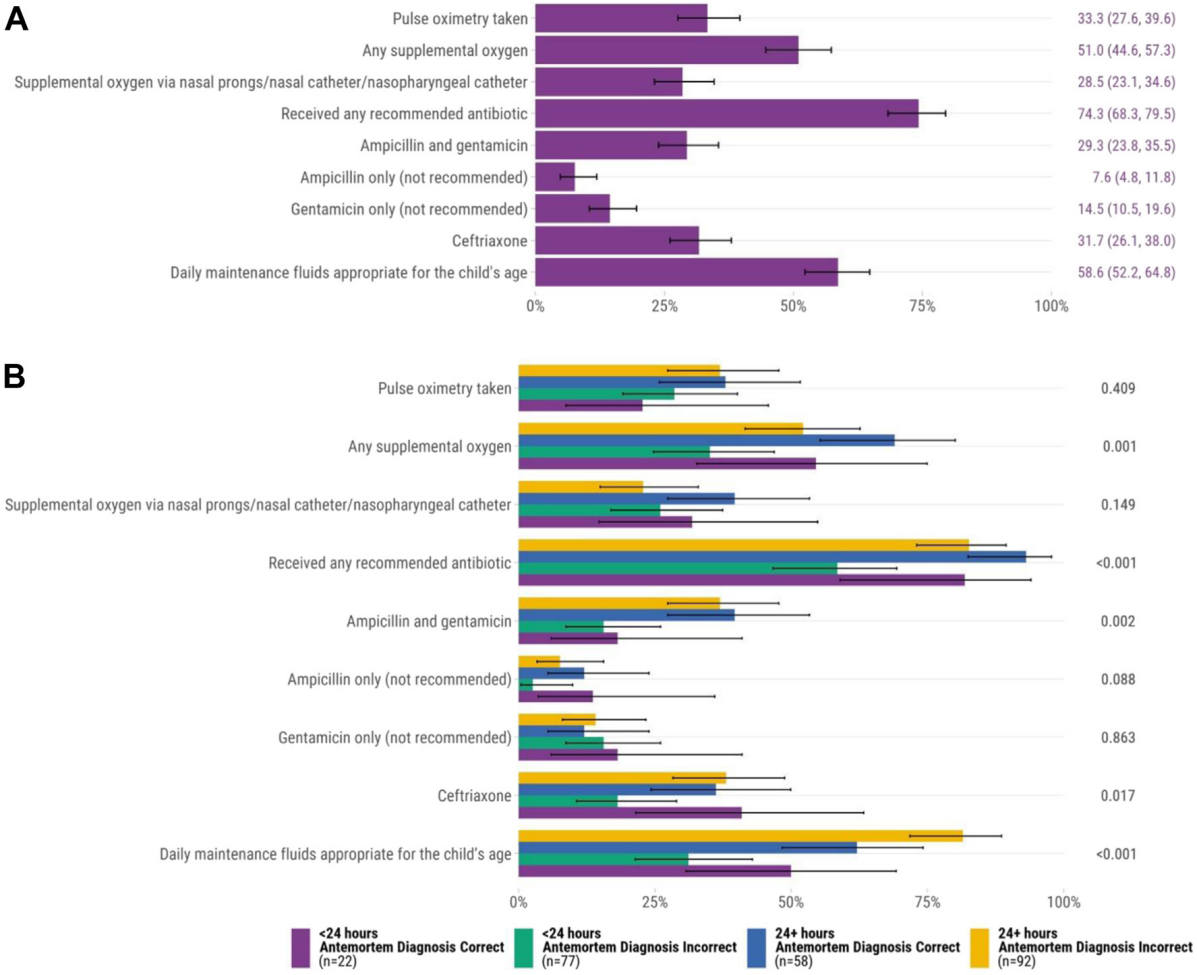
Adherence to WHO Pocket Book of Hospital Care for Children recommendations for lower respiratory infections determined by postmortem diagnosis (n = 249) A. All cases∗ B. Comparison of concordant vs. discordant antemortem and postmortem diagnoses and hospital admission duration∗∗ ∗Error bars represent 95% confidence intervals. ∗∗*P* values were calculated through chi-square test for each recommendation.

Among included infants and children, 249 (57.4%) had lower respiratory tract infections in the causal chain of mortality. Of these, 74.3% (n = 185) received any recommended antibiotic, including 29.3% (73/249) receiving ampicillin and gentamicin, 31.7% (79/249) receiving ceftriaxone (a WHO-recommended alternative therapy), and 10.0% (25/249) receiving both regimens (Fig. 1A). Although documentation of concomitant administration of supplemental oxygen when pulse oximetry was measured was not clear, according to records, 33.3% (n = 83) had pulse oximetry taken and 54.2% (n = 45) of those had oxygen saturation levels <90%, 6.0% (n = 5) had levels of 90–92%, and the remaining had oxygen saturation levels of 93–100%. Among all cases that had lower respiratory tract infections in the causal chain of mortality, 51.0% (n = 127) received any supplemental oxygen therapy. In addition, 57 (22.9%) infants and children had chest radiography performed.

Infants and children who had a concordant antemortem and postmortem diagnosis of lower respiratory tract infections and were admitted for ≥24 h had higher rates of administration of any supplemental oxygen, any recommended antibiotic, including ampicillin and gentamicin, and maintenance IV fluids compared to those admitted for <24 h with discordant antemortem and postmortem diagnoses (Fig. 1B). In unadjusted analyses, adherence to major diagnostics and therapeutics guideline adherence was highest at the site in Sierra Leone and lowest at the site in Mali (Supplemental Figure S1).

In multivariable analyses, age 1–11 months (adjusted odds ratio [aOR] 2.68, 95% confidence interval [CI] 1.30, 5.50), hospital admission of ≥24 h (aOR 4.89, 95% CI 2.06, 11.66), and having had concordant antemortem and postmortem diagnoses (aOR 3.32, 95% CI 1.38, 8.00) were independently associated with the administration of recommended antibiotics (Supplemental Table S2). Infants aged 1–11 months (aOR 2.38, 95% CI 1.32, 4.29) and those with concordant antemortem and postmortem diagnoses (aOR 2.42, 95% CI 1.28, 4.57) were more likely to receive any supplemental oxygen (Supplemental Table S3).

### Sepsis

**Fig. 2:**
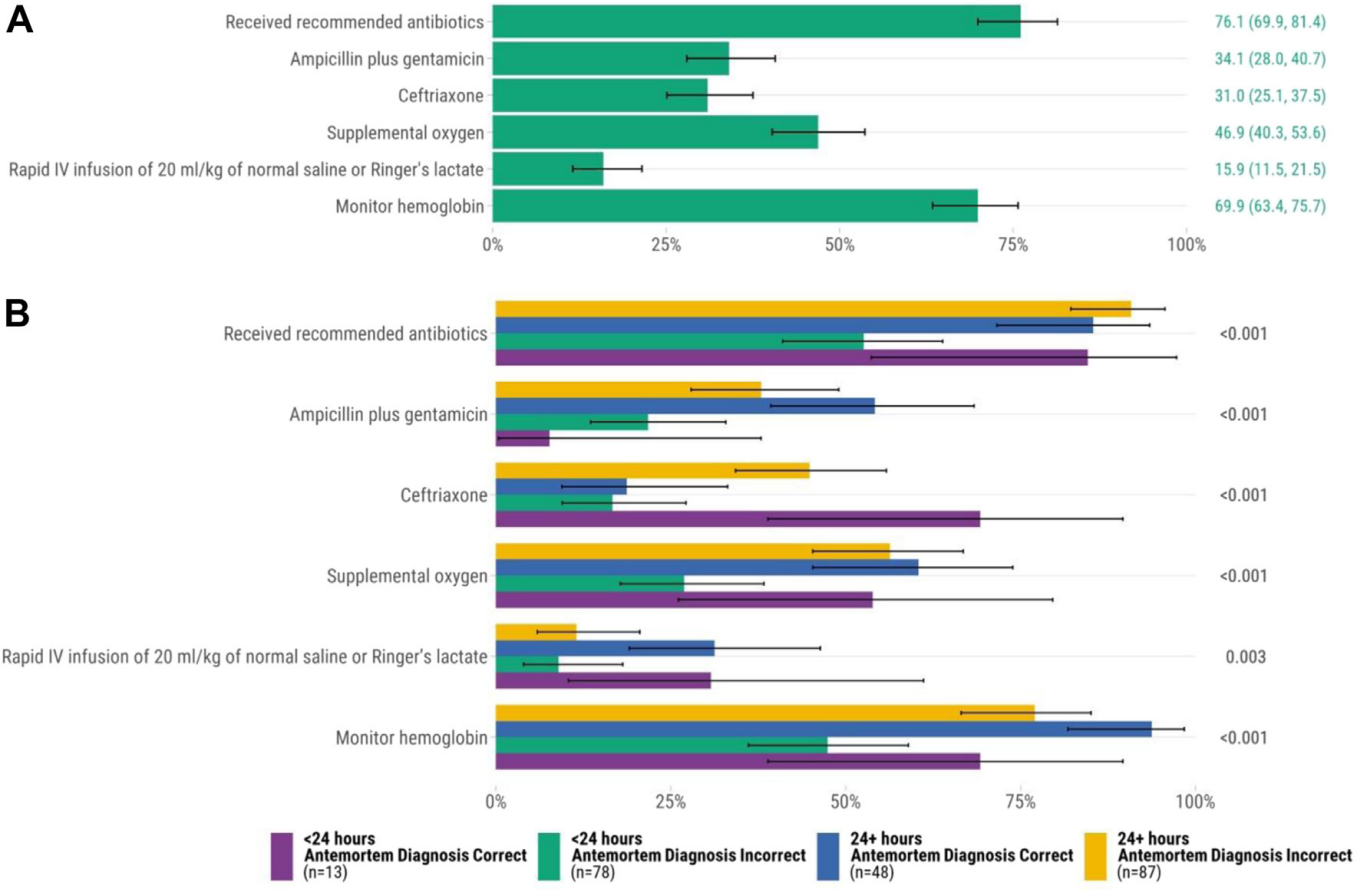
Adherence to WHO Pocket Book of Hospital Care for Children recommendations for sepsis determined by postmortem diagnosis (n = 226). A. All cases∗. B. Comparison of concordant vs. discordant antemortem and postmortem diagnoses and hospital admission duration∗∗. ∗Error bars represent 95% confidence intervals. ∗∗*P* values were calculated through chi-square test for each recommendation.

We assessed treatment for 226 infants and children who died from sepsis. Seventy-six percent (n = 172) received antibiotics during hospital admission. Of the 56 infants and children who died from sepsis and had documented shock before death (i.e., poor perfusion leading to end organ damage) 64.3% (n = 36) received intravenous (IV) fluids of either normal saline or Ringer's lactate rapidly at 20 mL/kg, per WHO recommendations (i.e., 15.9% of all sepsis-related deaths with and without documented shock) (Fig. 2A). Among the 56 infants and children who had sepsis and documented shock, 32.1% (n = 18) received vasopressors (i.e., adrenaline or dopamine), all of which also received IV fluids. Among the 72 cases that had both sepsis and malnutrition in the causal chain, 10 (13.9%) received IV fluids and five of these ten (50.0%) had documented shock. There were 69.9% (n = 158) infants and children with sepsis who had their hemoglobin monitored. There were 71 (31.4%) infants and children who had blood cultures drawn before their death. Among infants and children who died from sepsis who had concordant antemortem and postmortem diagnoses and whose hospital admissions were ≥24 h, there were higher rates of antibiotic administration, any supplemental oxygen, rapid infusion of IV fluids, and hemoglobin measurement than among those admitted for <24 h with discordant antemortem and postmortem diagnoses (Fig. 2B). Among infants and children who died of sepsis, we found no difference in the proportion who received IV fluids rapidly between the subset who also had malnutrition (n = 72) compared to those who did not (n = 154; *P* = 0.68). In unadjusted comparisons, adherence to WHO sepsis guidelines was higher at South Africa, Sierra Leone, Mozambique, and Ethiopia sites and lower in Mali (Supplemental Figure S2).

### Severe acute malnutrition

**Fig. 3:**
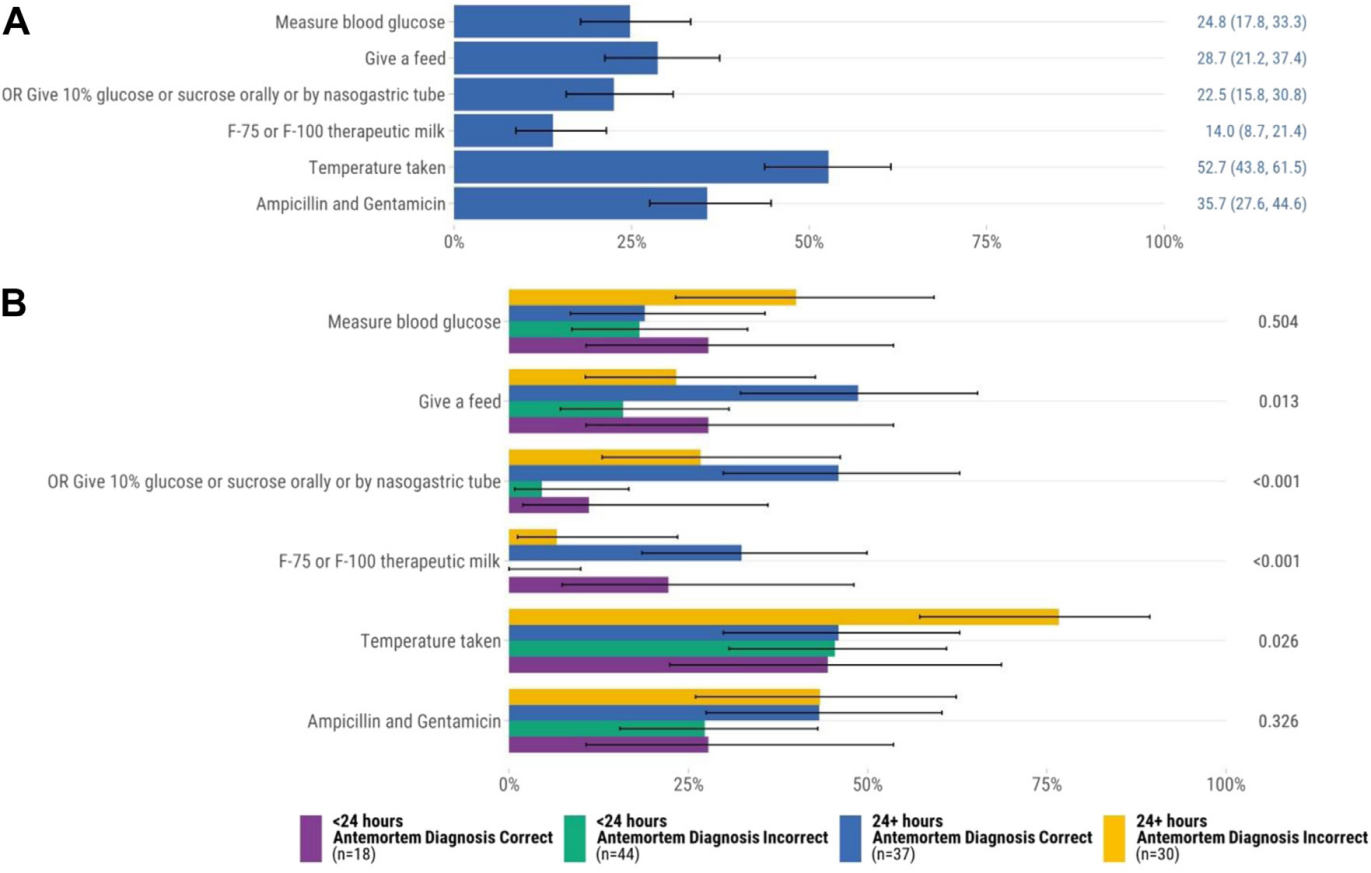
Adherence to WHO Pocket Book of Hospital Care for Children recommendations for severe acute malnutrition determined by postmortem diagnosis (n = 129). A. All cases∗. B. Comparison of concordant vs. discordant antemortem and postmortem diagnoses and hospital admission duration∗∗. ∗Error bars represent 95% confidence intervals. ∗∗*P* values were calculated through chi-square test for each recommendation.

One hundred twenty-nine infants and children had severe acute malnutrition in the causal chain of mortality. Of these, a minority had documentation of a blood glucose measurement (24.8%, n = 32) or received F-75 or F-100 therapeutic milk (14.0%, n = 18) per WHO recommendations (Fig. 3A). Among those with malnutrition who had a documented blood sugar (n = 17), four (36.3%) had a blood glucose of <54 mg/dL. Among those four, 50% (n = 2) did not receive F-75 or F-100 therapeutic milk. Over half had temperature documented (52.7%, n = 68). Approximately one in three received ampicillin and gentamicin (35.7%, n = 46). Among infants and children who had severe acute malnutrition in the causal chain, those who had a concordant antemortem diagnosis and were admitted for ≥24 h were more likely to receive feeds or the supplemental formulas F-75 or F-100 than those admitted for <24 h with discordant antemortem and postmortem diagnoses (Fig. 3B). The proportion of deceased infants and children who received guideline adherent treatment for severe acute malnutrition varied by site (Supplemental Figure S3).

### Malaria

**Fig. 4:**
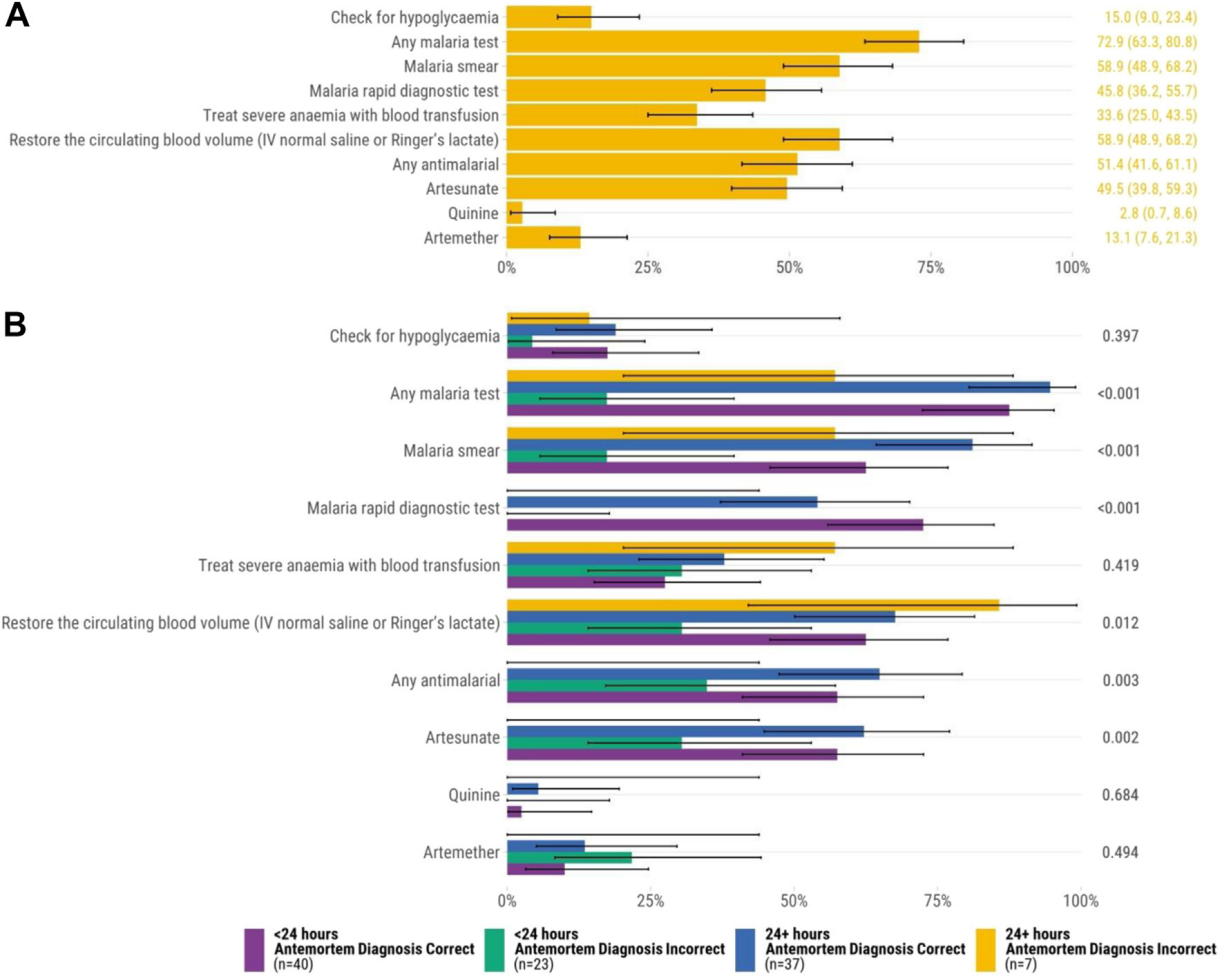
Adherence to WHO Pocket Book of Hospital Care for Children recommendations for malaria determined by postmortem diagnosis (n = 107). A. All cases∗. B. Comparison of concordant vs. discordant antemortem and postmortem diagnoses and hospital admission duration∗∗. ∗Error bars represent 95% confidence intervals. ∗∗*P* values were calculated through chi-square test for each recommendation.

There were 107 infants and children who had malaria in the causal chain of mortality. Of these, 72.9% (n = 78) had a documented antemortem malaria test and a minority had a blood glucose measurement documented (15.0%, n = 16). About half (51.4%, n = 55) received recommended antimalarial medications for severe malaria (artesunate [49.5%, n = 53] or artemether [13.1%, n = 14] were most common; Fig. 4A). There were 33.6% (n = 36) who received blood transfusions. Among infants and children who had malaria in the causal chain of mortality, those who had a concordant antemortem diagnosis and were admitted <24 h were more likely to have a malaria smear done than those admitted for <24 h with a discordant antemortem diagnosis (Fig. 4B). Infants and children who died from malaria and were malnourished (n = 39) were more likely to receive IV fluids rapidly than the 68 infants and children who died from malaria and were not malnourished (74% vs. 51%, *P* = 0.044). Adherence to other guideline recommendations for malaria did not differ by hospital admission duration or concordance of antemortem and postmortem diagnoses. Adherence to recommendations to provide blood transfusion for severe malaria and to administer antimalarials was more common in Sierra Leone than in Mozambique and Kenya (Supplemental Figure S4).

### Diarrheal diseases

**Fig. 5:**
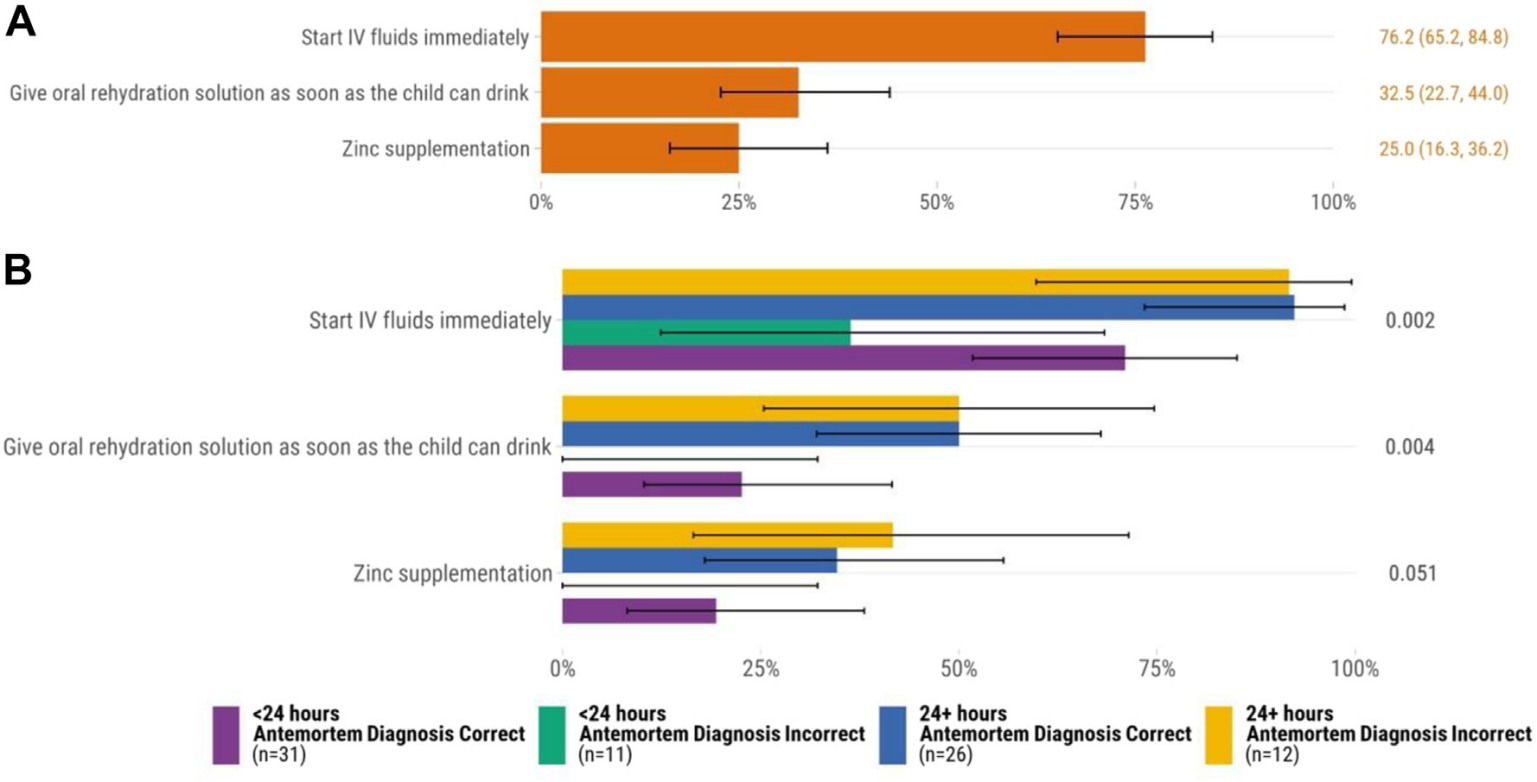
Adherence to WHO Pocket Book of Hospital Care for Children recommendations for diarrheal diseases determined by postmortem diagnosis (n = 80). A. All cases∗. B. Comparison of concordant vs. discordant antemortem and postmortem diagnoses and hospital admission duration∗∗. ∗Error bars represent 95% confidence intervals. ∗∗*P* values were calculated through chi-square test for each recommendation.

Eighty infants and children had diarrheal diseases in the causal chain of mortality. Of these, the majority received IV fluids (76.2%, n = 61) in accordance with the WHO recommendations (Fig. 5A). However, zinc supplementation was documented for 25.0% (n = 20) of patients. Regarding other recommended micronutrient supplementation among the 80 diarrheal deaths, 2 (2.5%) had record of vitamin A supplementation, 3 (3.8%) received folic acid, 5 (5.3%) received a multivitamin, 0 (0%) were given copper, and 0 (0%) received magnesium. No children with severe acute malnutrition and diarrheal disease had documented receipt of supplemental potassium. Infants and children who died from diarrheal diseases and had a concordant antemortem diagnosis and were admitted for ≥24 h had greater rates of IV fluid administration and oral rehydration solution than those with discordant antemortem and postmortem diagnoses and who were admitted <24 h (Fig. 5B). Administration of IV fluids was significantly less common among infants and children who died in Mali compared to the other sites (Supplemental Figure S5).

## Discussion

In our study of over 400 infants and children who died in seven sites with high childhood mortality rates, we aimed to assess provider adherence to WHO clinical care recommendations for the five leading causes of death among infants and children in high-mortality areas across seven countries. We found variable and often suboptimal adherence to the WHO recommendations for major causes of childhood mortality (i.e., lower respiratory tract infections, sepsis, severe acute malnutrition, malaria, and diarrheal diseases), all of which had been determined to be the causes of death using careful postmortem methods. However, adherence to recommendations was generally better among infants and children with concordant antemortem diagnoses than those with discordant antemortem and postmortem diagnoses. Moreover, clinical care recommendation adherence was generally higher among infants and children who had longer hospital admissions compared to those with shorter hospital admissions. Our findings shed light on the current status of clinical care and highlight areas that require improvement to reduce mortality rates in this vulnerable population.

The observed frequency of recommendation adherence for critical interventions such as antibiotics and supplemental oxygen for lower respiratory tract infections, fluid boluses for sepsis, antimalarial medications, and supplemental feeds for severe acute malnutrition among infants and children who died was lower than in prior observational studies that included patients who survived.^17^^,^^39^^,^^40^ Our findings differ from those in a study in Malawi that suggested that 25% of children who died from lower respiratory tract infections received supplemental oxygen.^41^ Moreover, prior studies in South Africa and Zambia demonstrated that 65–66% of children with lower respiratory tract infections received recommendation adherent antibiotic treatment, which is lower than we observed in our population of infants and children who died.^42^^,^^43^ Studies from the United States and Europe suggest varying adherence to guidelines for lower respiratory tract infections and sepsis in children, but have infrequently measured this among children who died.^44^, ^45^, ^46^ Our observational study cannot determine causation, but our findings raise the question of whether lower adherence occurs among infants and children who die. However, it is unclear if late treatment administration would be optimally beneficial or could have prevented the deaths included in our analyses. As multiple causes of death are common in CHAMPS, we could not account for which diagnoses and recommendations were prioritized by clinicians. A prospective study to assess clinician prioritization of treatment in settings with limited resources may be warranted.

Moreover, it should be noted that findings from a multicenter trial in Uganda, Kenya, and Tanzania suggest greater mortality among children with impaired perfusion due to severe infection (including children who met clinical criteria for sepsis) who received rapidly administered fluids than those who did not.^47^ Thus, adherence to this recommendation may have been influenced by the current controversy that exists regarding the WHO recommendation to rapidly administer IV fluids to children with sepsis. Additionally, only one in three infants and children who had documented shock received vasopressors. Additional study is warranted to elucidate clinician decision-making and resource constraints that may contribute to the administration of vasopressors. Children with severe malnutrition were excluded from the multicenter trial in Uganda, Kenya, and Tanzania of IV fluids^47^ and the WHO Pocket Book of Hospital Care for Children recommends IV fluids be given only to children with severe acute malnutrition who have signs of shock.^13^

Our findings point to the treatments that are infrequently administered to infants and children (i.e., any supplemental oxygen, administration of supplemental formula for severe acute malnutrition, etc.) in sites with high rates of childhood mortality. Multifaceted interventions that include sharing copies of recommendations, focused training, job aides, and feedback to providers have shown greater adherence to recommendations for the clinical care of children.^48^ Such approaches may be implemented to improve clinical care recommendation adherence. Additionally, results from a randomized controlled trial conducted in Guinea-Bissau that included >950 infants and children suggest that adherence to clinical care recommendations reduces inpatient mortality for malaria.^49^ Thus, additional efforts to encourage clinical care recommendation adherence are needed in regions with high childhood mortality.

The greater recommendation adherence in cases where the antemortem diagnosis was concordant with the postmortem diagnosis we observed suggests the need for more widely available and accurate diagnostics to arrive at the correct diagnosis. A previous study in Mozambique indicated a similar association between correct clinical diagnoses and recommendation-adherent administration of therapeutic measures.^50^ Laboratory and radiographic capacity are inadequate in resource-limited settings,^51^, ^52^, ^53^ which may have contributed to misdiagnoses among the infants and children included here. Efforts are urgently needed to expand diagnostic capacity and clinical skills in resource-limited settings to enhance diagnostic accuracy, as this may improve clinical care recommendation adherence.

We observed greater adherence to recommendations among infants and children with hospital admissions lasting ≥24 h than those whose death occurred within 24 h of admission. This link to time in the hospital is likely due to several factors including late presentation, needed time to obtain medications or labs, and diagnostic clarity that may come with time during hospital admission. As much as 60% of deaths among hospitalized children occur within 24 h of admission in resource-limited settings.^54^, ^55^, ^56^ Thus, efforts are needed to improve early diagnosis and to improve resuscitative efforts to prevent inpatient mortality among children. Such initiatives have shown reductions in inpatient mortality rates in Malawi and Ghana.^57^^,^^58^ Additionally, studies are needed to better understand the reasons for delays in diagnostics and therapeutics early during hospital admission.

This study should be interpreted in the context of its limitations. The CHAMPS network does not collect data on infants and children who survive, which precludes our ability to measure the impact of recommendation adherence on mortality reduction. Future studies to assess potential differences in recommendation adherence among children who survive and those who die are thus warranted. Additionally, due to the retrospective nature of this study, we relied on clinical data documented by treating clinicians and nurses. Thus, it is possible that some components of clinical care were provided but not documented, leading to over-estimation of gaps in clinical care for common causes of death in the CHAMPS network. Moreover, we did not measure documented indications for medications or IV fluids, dosages of administered medications, and the frequency of therapeutic milk feeds for malnutrition, so we cannot account for inappropriate administration based on clinical presentation, potential under- or over-dosing of medications, IV fluids, or therapeutic milk administered to infants and children who died. Although CHAMPS includes seven sites in sub-Saharan Africa and South Asia, our findings may not represent recommendation adherence in other regions or in the same regions with differing resources within healthcare facilities. Moreover, some sites contributed fewer cases than others, owing to differences in enrollment rates in CHAMPS.

Our findings also do not apply to community deaths among infants and children who were not hospitalized before their death. Additional studies are warranted to assess clinical care administered, if any, in cases that die in the community without antecedent hospitalization. We relied on available variables that were included in our multivariable models. However, there may be other unmeasured confounding variables such as socioeconomic factors (i.e., caregiver ability to pay for clinical care, urbanicity of residence, etc.) that may provide additional insight into factors associated with the administration of key therapeutics. Lastly, we were unable to determine if recommended diagnostics and therapeutics were not provided due to alternate reasons such as a lack of provider recognition of disease, due to medication shortages, infrastructure/supply issues leading to unavailable resources, or clinician suspicion for alternate diagnoses such as a viral illness in lower respiratory tract infections in which case antibiotic administration may not have been warranted as recommended in WHO guidelines. Further study is needed to better elucidate the reasons for the under-administration of many diagnostics and therapeutics in our study. The findings and conclusions in this report are those of the authors and do not necessarily represent the views of the US Centers for Disease Control and Prevention.

In our study to assess provider adherence to WHO clinical care recommendations for leading causes of death among infants and children in high-mortality areas across seven countries, we found suboptimal adherence to WHO recommendations for lower respiratory tract infections, sepsis, severe acute malnutrition, malaria, and diarrheal diseases among infants and children. Adherence was generally better, however, among children who were correctly diagnosed in the antemortem period and among children who were admitted for ≥24 h. Studies are urgently needed to understand the reasons for gaps in clinical care recommendation adherence in sub-Saharan Africa and Bangladesh to, in turn, develop clinical and health system level interventions to reduce gaps in clinical care recommendation adherence.

## Contributors

CGW secured the funding. CAR, KAI, IO, and CGW conceptualized and designed the study and interpreted the data. CAR wrote the first draft of the manuscript. ZJM and CAR verified the underlying data and conducted the statistical analyses. KAI, VA, DO, SEA, ESG, MZH, AR, MA, JAGS, NA, LM, AB, HL, KLK, SOS, MDT, AMK, DS, AS, RV, SA, QB, IM, BATP, IO, CJC, I-AB, RL, KG, SM, ZD, SM, SV, JDT, PCM, DMB, and RFB oversaw data collection, verified the underlying data, assisted with the interpretation of the data, and reviewed and provided input to the final draft. All authors read and approved the final version of the manuscript. All authors had full access to all the data in the study and accept responsibility to submit for publication.

## Data sharing statement

The dataset, data dictionary generated during the study, the statistical plan, and analytic code may be available from the corresponding author upon reasonable request after all planned manuscripts have been accepted for publication. If requested, data will include de-identified patient information. If requested, we will make the data without identifiers available under a data-sharing agreement inasmuch as the data are only used for research purposes, along with a commitment to destroy or return the data after analyses are complete.

## Declaration of interests

CGW received honoraria from the University of St. Andrews for speaking to alumni about CHAMPS and global health work. JAGS reports receiving funding from the Wellcome Trust, UK FCDO, European Union, and the National Institute for Health Research. SM has received grants from the Bill & Melinda Gates Foundation, GSK, Pfizer, Minervax, Novavax, Providence, Gritstone, and ImmunityBio. SM has received honoraria from GSK for lecturing. CGW and SM report serving on data safety monitoring boards for SPEAC (CGW) and PATH and CAPRISA (SM). All other investigators declare no competing interests.

## Appendix A Supplementary data

Supplemental Tables

CHAMPS Consortium Members
