# Supplementary material for: Provider adherence to clinical care recommendations for infants and children who died in seven low- and middle-income countries in the Child Health and Mortality Prevention Surveillance (CHAMPS) network
Source: eClinicalMedicine. 2023 Aug 31;63:102198. doi: 10.1016/j.eclinm.2023.102198 (PMC10484959; doi:10.1016/j.eclinm.2023.102198)
Supplement: Supplemental Tables [file mmc1.docx]

**Supplemental Table 1.** Comparison of deceased infants and children aged 1-59 months in the CHAMPS network included and excluded in the analysis of clinical care recommendation adherence

|  | **Included in Analyses (N=460), n (%)** | **Excluded from Analyses (N=191), n (%)** | ***P* value** |
| --- | --- | --- | --- |
| **Patient Age, month (median, [IQR])** | 12 (4, 23) | 9 (4, 21) | 0∙477 |
| **Sex** |  |  | 0∙667 |
| Male | 244 (53∙0) | 105 (55∙0) |  |
| Female | 216 (47∙0) | 86 (45∙0) |  |
| **Causes of Death** |  |  |  |
| Lower respiratory tract infections | 249 (54∙1) | 58 (30∙4) | <0∙001 |
| Sepsis | 226 (49∙1) | 56 (29∙3) | <0∙001 |
| Severe Acute Malnutrition | 129 (28∙0) | 43 (22∙5) | 0∙172 |
| Malaria | 107 (23∙3) | 40 (20∙9) | 0∙539 |
| Diarrheal Disease | 80 (17∙4) | 21 (11∙0) | 0∙043 |

**Supplemental Table 2.** Factors associated with the administration of any guideline-adherent antibiotic for lower respiratory tract infections (N=235^a^)

|  | **Therapeutic Yes, n (%)**  **N = 181** | **Therapeutic No, n (%)**  **N = 54** | **Odds Ratio (95% CI)^b^** | ***P* value** | **Adjusted Odds Ratio (95% CI)^c^** | ***P* value** |
| --- | --- | --- | --- | --- | --- | --- |
| **Age at the time of death** |  |  |  | 0∙002 |  | 0∙007 |
| 1-11 months | 111 (61∙3) | 22 (40∙7) | 2∙82 (1∙44, 5∙52) |  | 2∙68 (1∙30, 5∙50) |  |
| 12-59 months | 70 (38∙7) | 32 (59∙3) | *Referent* |  | *Referent* |  |
| **Sex** |  |  |  | 0∙809 |  | – |
| Male | 99 (54∙7) | 30 (55∙6) | *Referent* |  | – |  |
| Female | 82 (45∙3) | 24 (44∙4) | 1∙08 (0∙57, 2∙05) |  | – |  |
| **Time from admission to death^d^** |  |  |  | <0∙001 |  | <0∙001 |
| <24 hours | 53 (29∙3) | 33 (61∙1) | *Referent* |  | *Referent* |  |
| ≥24 hours | 128 (70∙7) | 21 (38∙9) | 5∙54 (2∙45, 12∙53) |  | 4∙89 (2∙06, 11∙66) |  |
| **Concordant antemortem and postmortem diagnoses^d^** |  |  |  | 0∙002 |  | 0∙008 |
| Yes | 72 (39∙8) | 8 (14∙8) | 3∙60 (1∙58, 8∙19) |  | 3∙32 (1∙38, 8∙00) |  |
| No | 109 (60∙2) | 46 (85∙2) | *Referent* |  | *Referent* |  |

^a^ Admission diagnosis was not available for 14 deaths from lower respiratory tract infections, which were excluded from analysis. Variance inflation factors between independent variables were all <1.003 so there was no evidence of collinearity.

^b^ All values are in mixed effects model controlled for random effect of site.

^c^ All values are in mixed effects model controlled for fixed effects for variables in column and random effect for site.

^d^ Interaction term between admission time and concordant diagnosis was not statistically significant (p=0∙983).

**Supplemental Table 3.** Factors associated with the administration of any supplemental oxygen for lower respiratory tract infections (N=235^a^)

|  | **Therapeutic Yes, n (%)**  **N = 126** | **Therapeutic No, n (%)**  **N = 109** | **Odds Ratio (95% CI)^b^** | ***P* value** | **Adjusted Odds Ratio (95% CI)^c^** | ***P* value** |
| --- | --- | --- | --- | --- | --- | --- |
| **Age at the time of death** |  |  |  | 0∙004 |  | 0∙004 |
| 1-11 months | 84 (66∙7) | 49 (45∙0) | 2∙32 (1∙31, 4∙09) |  | 2∙38 (1∙32, 4∙29) |  |
| 12-59 months | 42 (33∙3) | 60 (55∙0) | *Referent* |  | *Referent* |  |
| **Sex** |  |  |  | 0∙170 |  | 0∙220 |
| Male | 73 (57∙9) | 56 (51∙4) | *Referent* |  | *Referent* |  |
| Female | 53 (42∙1) | 53 (48∙6) | 0∙68 (0∙39, 1∙18) |  | 0∙69 (0∙38, 1∙25) |  |
| **Time from admission to death** |  |  |  | 0∙868 |  | – |
| <24 hours | 39 (31∙0) | 47 (43∙1) | *Referent* |  | – |  |
| ≥24 hours | 87 (69∙0) | 62 (56∙9) | 1∙06 (0∙55, 2∙02) |  | – |  |
| **Concordant antemortem and postmortem diagnoses** |  |  |  | 0∙010 |  | 0∙006 |
| Yes | 52 (41∙3) | 28 (25∙7) | 2∙20 (1∙20, 4∙03) |  | 2∙42 (1∙28, 4∙57) |  |
| No | 74 (58∙7) | 81 (74∙3) | *Referent* |  | *Referent* |  |

^a^ Admission diagnosis was not available for 14 deaths from lower respiratory tract infections, which were excluded from analysis. Variance inflation factors between independent variables were all <1.010 so there was no evidence of collinearity.

^b^ All values are in mixed effects model controlled for random effect of site.

^c^ All values are in mixed effects model controlled for fixed effects for variables in column and random effect for site

**Supplemental Figure 1.** Adherence to WHO Pocket Book of Hospital Care for Children recommendations for lower respiratory infections determined by postmortem diagnosis (N=249) by site*

**
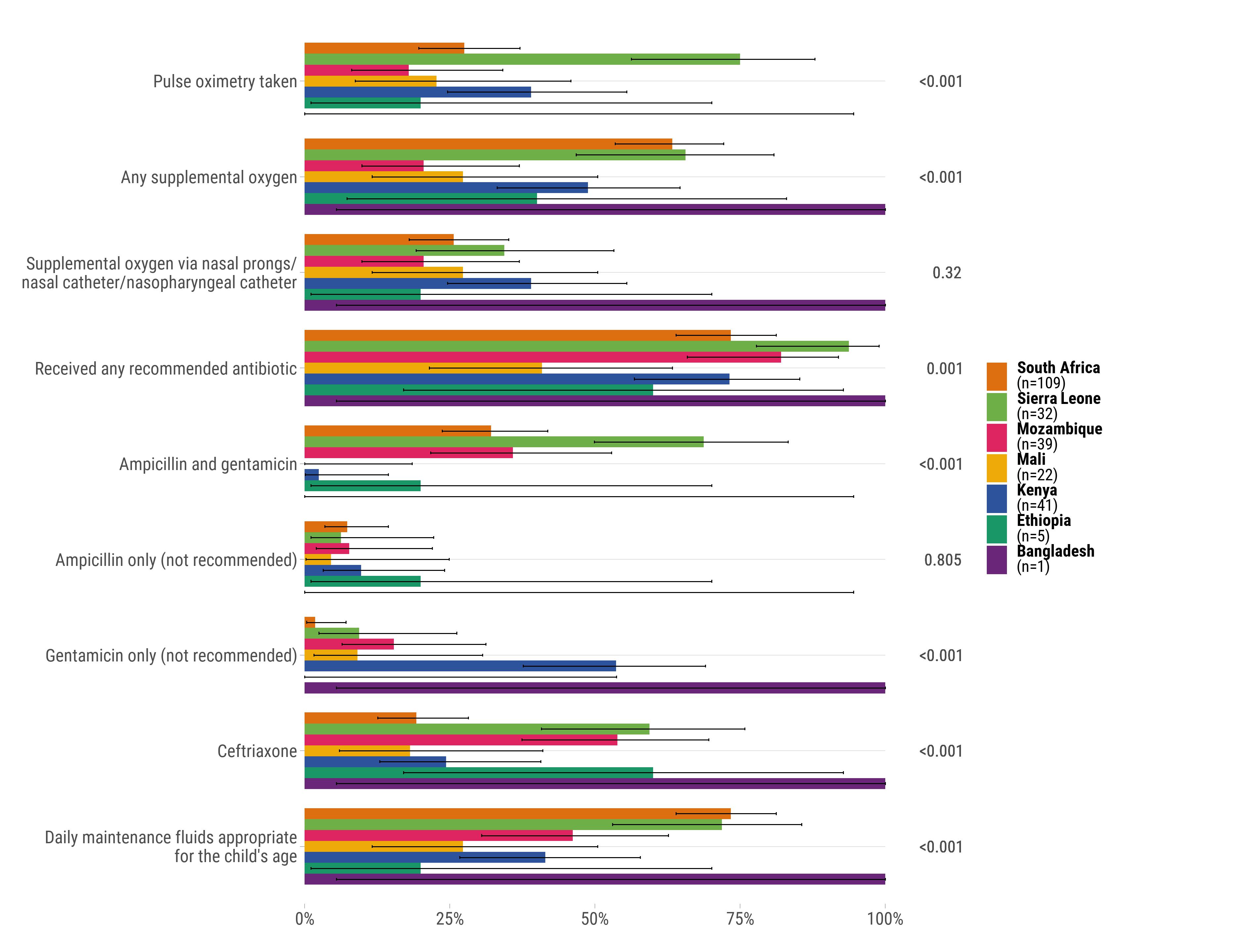
**

*Error bars represent 95% confidence intervals. *P* values were calculated through chi-square test for each recommendation.

**Supplemental Figure 2**. Adherence to WHO Pocket Book of Hospital Care for Children recommendations for sepsis determined by postmortem diagnosis (N=226) by site


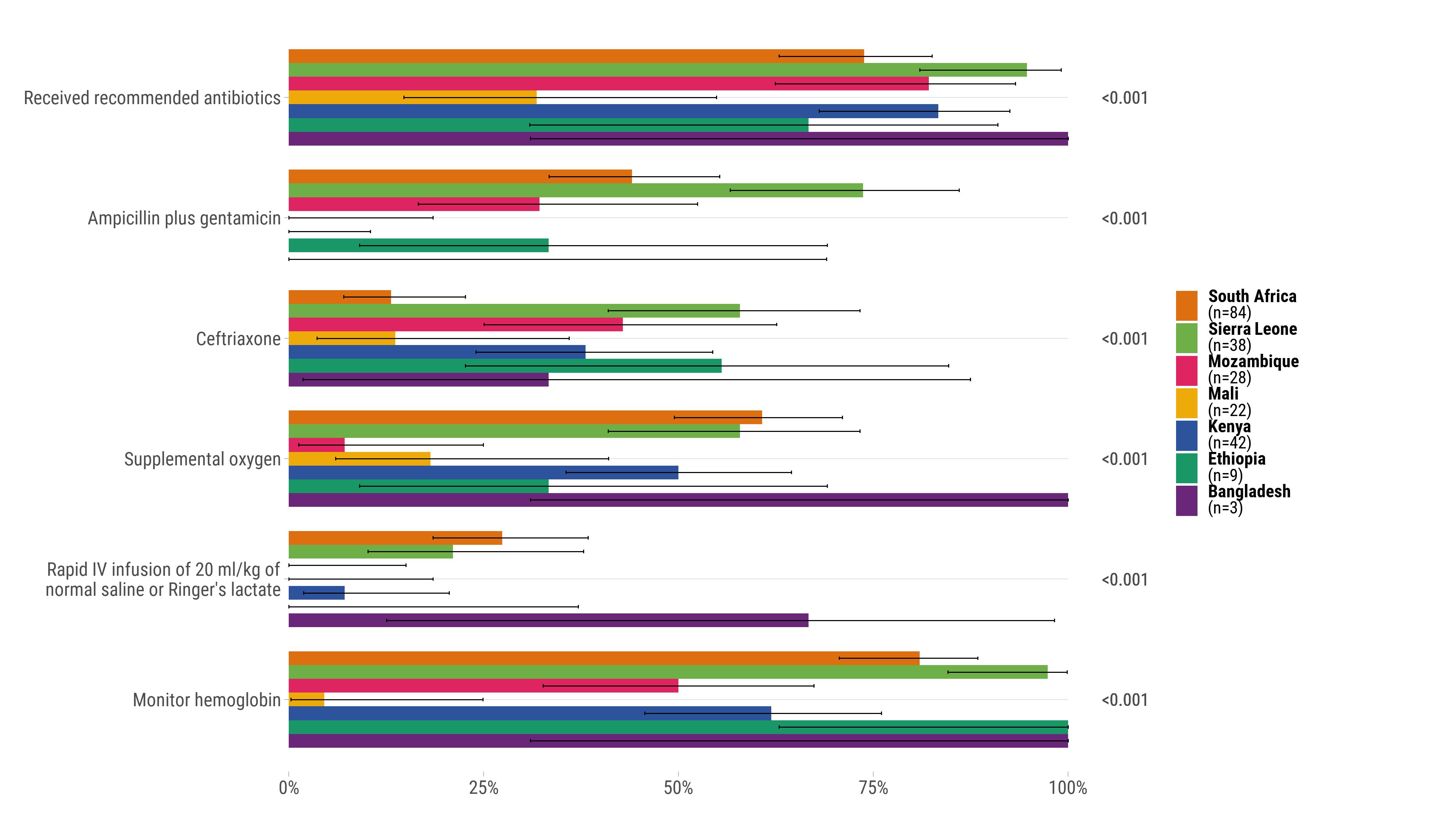


*Error bars represent 95% confidence intervals. *P* values were calculated through chi-square test for each recommendation.

**Supplemental Figure 3**. Adherence to WHO Pocket Book of Hospital Care for Children recommendations for malnutrition determined by postmortem diagnosis (N=129) by site

**
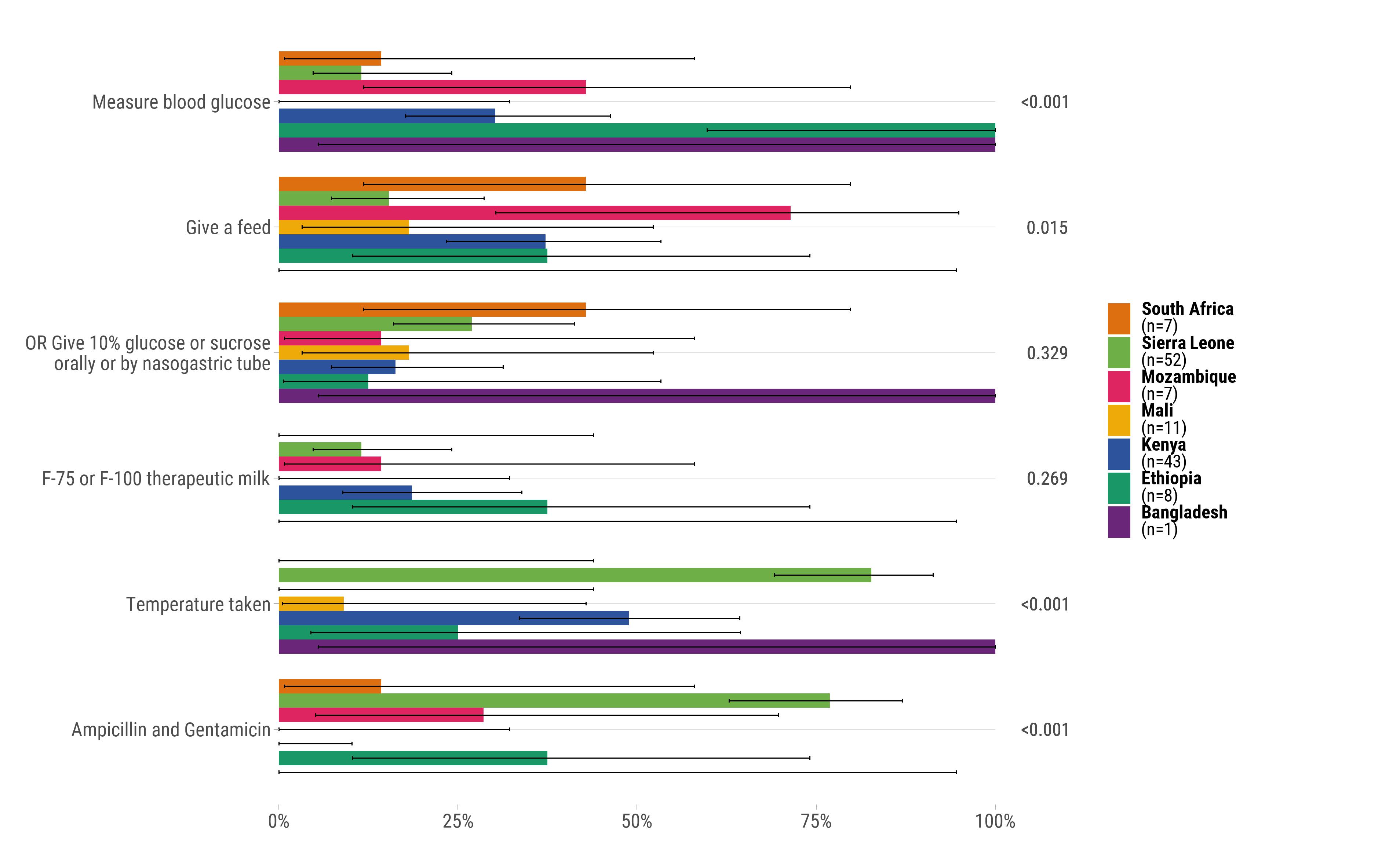
**

*Error bars represent 95% confidence intervals. *P* values were calculated through chi-square test for each recommendation.

**Supplemental Figure 4**. Adherence to WHO Pocket Book of Hospital Care for Children recommendations for malaria determined by postmortem diagnosis (N=107) by site

**
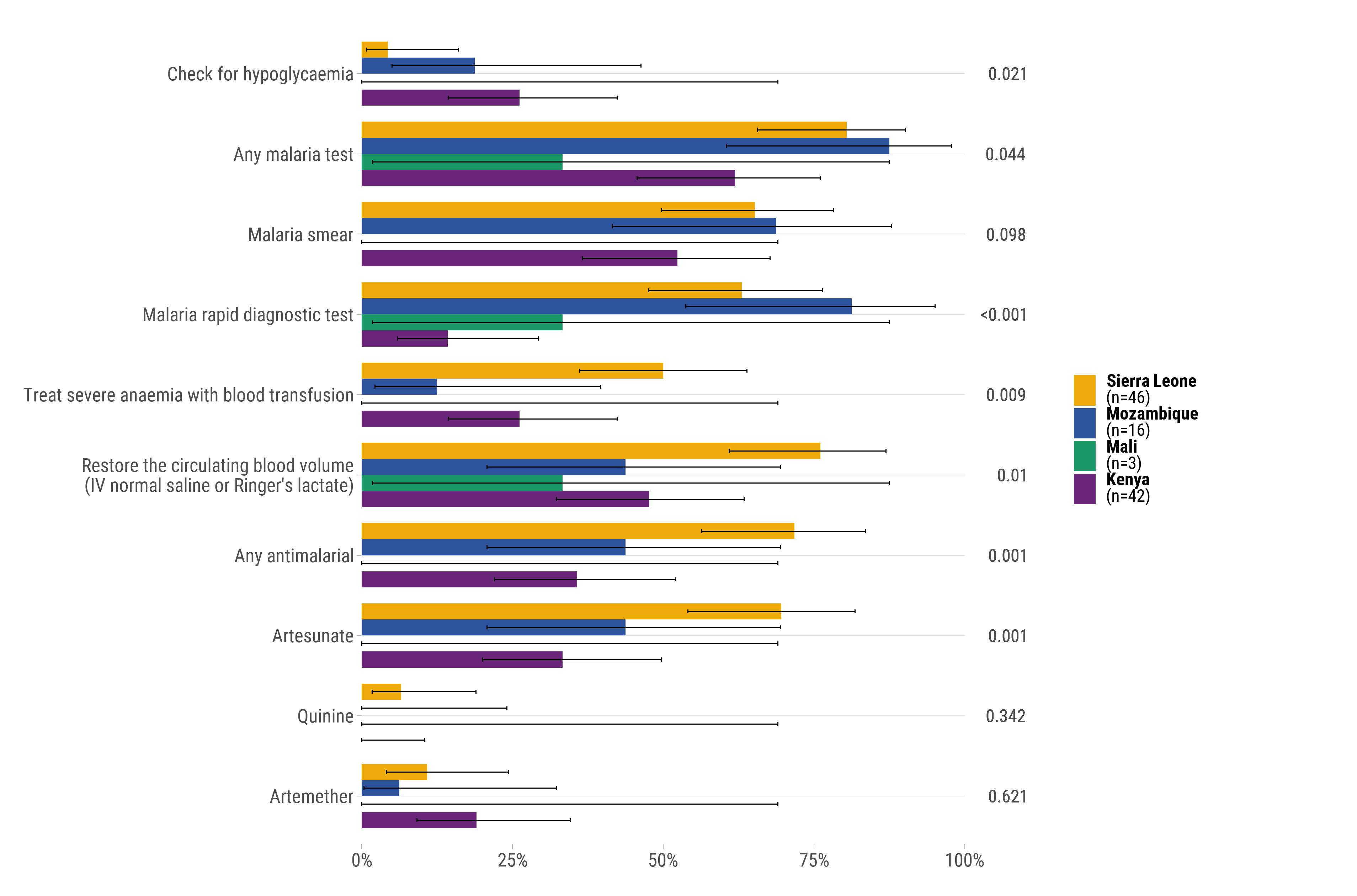
**

*Error bars represent 95% confidence intervals. *P* values were calculated through chi-square test for each recommendation.

**Supplemental Table 5**. Adherence to WHO Pocket Book of Hospital Care for Children recommendations for diarrheal diseases determined by postmortem diagnosis (N=80) by site


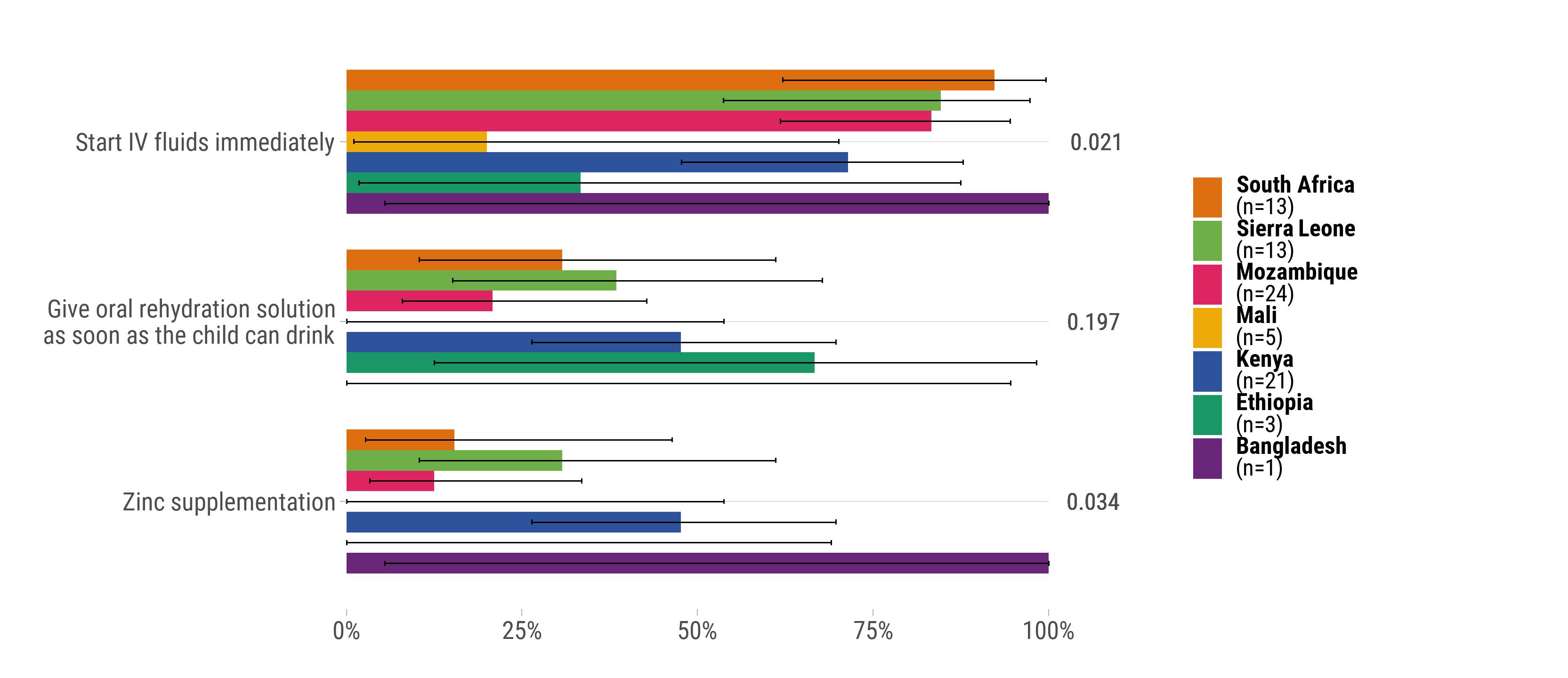


*Error bars represent 95% confidence intervals. *P* values were calculated through chi-square test for each recommendation.
